# The LINC00152/miR-205-5p/CXCL11 axis in hepatocellular carcinoma cancer-associated fibroblasts affects cancer cell phenotypes and tumor growth

**DOI:** 10.1007/s13402-022-00730-4

**Published:** 2022-11-26

**Authors:** Gao Liu, Zhang-Fu Yang, Jian Sun, Bao-Ye Sun, Pei-Yun Zhou, Cheng Zhou, Ruo-Yu Guan, Zhu-Tao Wang, Yong Yi, Shuang-Jian Qiu

**Affiliations:** Department of Liver Surgery and Transplantation, Liver Cancer Institute and Biomedical Research Center, Zhongshan Hospital, Fudan University, 180 Fenglin Road, 200032 Shanghai, People’s Republic of China

**Keywords:** Hepatocellular carcinoma (HCC), Cancer-associated fibroblasts (CAFs), LINC00152, MiR-205-5p, CXCL11

## Abstract

**Background:**

CXCL11 has been reported to be up-regulated in hepatocellular carcinoma (HCC) tissues and cancer-associated fibroblasts (CAFs), and CAF-secreted CXCL11 has been found to promote HCC cell proliferation and migration. Knowledge on how CAFs promote HCC progression is imperative for the future design of anti-tumor drugs addressing the high rates of disease recurrence. Herein, we propose a mechanism by which LINC00152 positively regulates CXCL11 expression and, subsequently, HCC cell phenotypes and growth characteristics via miR-205-5p in CAFs.

**Methods:**

The expression of LINC00152, miR-205-5p in HCC/non-cancerous tissues, CAFs/NFs and HCC cell lines was determined by RT-qPCR. The CXCL11 expression and secretion were determined by westernblot and ELISA. Different expressions of LINC00152, CXCL11 and miR-205-5p in CAFs were achieved by transfection with corresponding overexpression/knockdown vectors or mimics/inhibitor. The interactions among LINC00152, miR-205-5p and CXCL11 were confirmed by FISH, luciferase, AGO2 and RNA-pulldown assays. Transwell, colony formation and MTT assays were performed to assess the role of CAFs conditioned medium (CM) in HCC cell phenotype. BALB/c nude mice xenografts were used to determine the role of CAFs on HCC growth *in vivo*.

**Results:**

We found that in vitro, CM from CAFs transfected with sh-LINC00152 dramatically suppressed HCC cell viability, colony formation and migration, and that CM from CAFs transfected with miR-205-5p inhibitor (CAF-CM (miR-205-5p inhibitor)) exerted opposite effects on HCC cell phenotypes. Exogenous overexpression of CXCL11 in CAFs or CAF-CM (miR-205-5p inhibitor) could partially attenuate the effects of LINC00152 knockdown. In contrast, CM from CAFs transfected with LINC00152 dramatically increased HCC cell viability, colony formation and migration, and CM from CAFs transfected with miR-205-5p mimics (CAF-CM (miR-205-5p mimics)) exerted opposite effects on HCC cell phenotypes. Knockdown of CXCL11 in CAFs or CAF-CM (miR-205-5p mimics) could partially attenuate the effects of LINC00152 overexpression. *In vivo*, LINC00152 knockdown in CAFs inhibited tumor growth in a mouse model, which could be reversed by CXCL11 overexpression in CAFs. Mechanistically, we found that LINC00152 could act as a ceRNA to counteract miR-205-5p-mediated suppression on CXCL11 by directly binding to miR-205-5p and the 3’UTR of CXCL11.

**Conclusion:**

Our data indicate that a LINC00152/miR-205-5p/CXCL11 axis in HCC CAFs can affect the proliferative and migrative abilities of HCC cells in vitro and HCC tumor growth *in vivo*.

**Supplementary Information:**

The online version contains supplementary material available at 10.1007/s13402-022-00730-4.

## Introduction

Liver cancer is one of the top causes of cancer-related death worldwide, and about 90% of primary hepatic malignant tumors are hepatocellular carcinoma (HCC) [1, 2]. Unlike most other malignancies, almost all HCCs arise after chronic liver inflammation and cirrhosis [3]. Regardless of the etiology, HCC develops as a result of a series of typical liver changes: chronic hepatic necrosis-inflammation, compensatory hyperplasia, liver fibrosis and subsequent cirrhosis. Further exploration of the potential mechanisms by which chronic inflammation in the tumor microenvironment affects HCC development is of great importance for the development of treatment regimens for HCC.

Cancer-associated fibroblasts (CAFs) are a dominant cell type within the reactive stroma of HCC and the major source of extracellular matrix (ECM) after hepatic stellate cells (HSCs) are activated into myofibroblasts [4, 5]. By remodeling the tumor microenvironment and the paracrine production of various cytokines, CAFs can promote proliferative, migrative and invasive abilities, EMT (epithelial-mesenchymal transformation), resistance to treatment and the acquisition of CSC (cancer stem cell)-like phenotypes of HCC cells [6, 7]. Consequently, CAFs can promote the development and metastasis of HCC through cross-talk with cohabitating cancer cells [8, 9]. For instance, Lin et al. previously reported that CAF-regulated genes, CCL2, CCL26, IL6, and LOXL2, may promote the proliferation, migration and invasion of HCC cells [10]. Jia et al. [11] also showed that CAFs can promote the proliferation of HCC cells by secreting HGF. Besides, Jiang et al. showed that peri-tumor associated fibroblasts may also play a significant role in tumor progression by recruiting tumor stem cells, maintaining the characteristics of stem cells and enhancing intrahepatic HCC metastasis by secreting IL-6, CXCL1, CCL2, SCGF-β, CXCL8, HGF and some other cytokines [12]. More importantly, we previously showed that CXCL11/CXCR3 is present in HCC tissues, particularly in high-metastatic HCC tissues and that CAF-secreted CXCL11 promoted HCC cell migration and metastasis [13]. Thus, CAF-secreted CXCL11 may mediate the interaction between CAFs and their cohabitating cancer cells during HCC progression.

Long non-coding RNAs (lncRNAs) are a type of non-coding RNAs (ncRNAs), defined as transcripts with lengths exceeding 200 nucleotides that are not translated into protein [14]. Growing evidence indicates that LncRNAs play critical roles in biological activities that underly the pathological physiology of multiple human diseases, such as inflammation and neoplasia [15, 16], chronic liver diseases [15, 16] as well as HCC [17, 18]. Several lncRNAs, including PDIA3P1, TSLNC8, DILC and LINC00665, have been shown to regulate IL-6/STAT3 or NF-κB signaling and to play a role in drug resistance and hepatic cancer progression [19–22]. For example, MALAT1 has been found to recruite BRG1, a catalytic subunit of the chromatin remodeling complex switching/sucrose non-fermentable (SWI/SNF) to the promoter regions of IL-6 and CXCL8, thereby promoting NF-κB-induced expression of these inflammatory factors [23]. The application of next-generation sequencing and microarray analysis has advanced our understanding of lncRNAs associated with multiple disease types [24], and HCC-related lncRNAs have been shown to play significant roles in HCC occurrence, development and repression [25]. Thus, we speculated that CAF-related lncRNAs may also contribute to HCC cell phenotype control.

LncRNAs may function as critical cis- or trans-acting modulators of gene activities through multiple mechanisms. For example, lncRNAs can competitively bind to miRNAs to act as competing endogenous RNAs (ceRNAs) and, thus, reverse the miRNA-rendered effects on downstream targets [26, 27]. Through this ceRNA mechanism, miRNAs and lncRNAs exert important but opposite effects on modulating gene expression through their fine tuning at different levels of transcription and translation which, in turn, may affect the invasion, metastasis, drug resistance and radioresistance of HCC cells [28–30]. Therefore, we started with the analysis of previous whole human genome microarray data to identify differentially expressed lncRNAs regulating CXCL11 in CAFs. We found that LINC00152 may be of potential interest. Specific effects of LINC00152 knockdown or co-effects of LINC00152 and CXCL11 in CAFs on HCC cell phenotypes and tumor growth in mice were examined. Using prediction tools, we found that miR-205-5p may target LINC00152 and CXCL11. The predicted binding was verified, and the co-effects of the LINC00152/miR-205-5p axis in CAFs on CXCL11 and HCC cell phenotypes were experimentally tested.

## Materials and methods

### Clinical sample collection

A total of 16 cases of invasive HCC tissues and adjacent non-cancerous tissues were collected immediately after surgical resection from Zhongshan Hospital, Fudan University. All cases were pathologically confirmed as HCC and none of the patients received either preoperative radiotherapy or chemotherapy. Informed consent was signed and obtained from each patient. All the clinical sampling was performed with approval of the Ethics Committee of the Zhongshan Hospital, Fudan University. Immediately after sample harvest, tissues were stored at -80 °C for further use.

### Isolation and characterization of CAFs and NFs from HCC samples

Fresh HCC specimens and corresponding adjacent non-cancerous tissues were washed with serum-free DMEM/F-12 medium, cut into 0.2 × 0.2 mm fragments, and cultured in fresh medium for 24 h for attachment. At the end of the incubation period, the unattached cells were removed and the attached cells were grown in the culture dishes for 2–3 weeks. The culture medium was replenished every two or three days until fibroblasts began to grow out. To verify isolated CAFs and normal fibroblasts (NFs), expression of the fibroblast markers α-SMA and FAP was examined by immunofluorescence staining [13]. CAFs and NFs should be α-SMA- and FAP-positive but CD31-, AFP- and pan-cytokeratin-negative. CAFs and NFs of less than ten generations are used for the experiments.

### Real-time quantitative polymerase chain reaction (RT-qPCR)

According to the kit protocols, total RNA was extracted from cells and tissues (TOYOBO, Tokyo, Japan). PCR-based analyses were performed following methods as previously described [13] using a universal SYBR Green Master System (Roche, Basel, Switzerland). Relative expression levels were calculated using the 2^−ΔΔCt^ method. The primers used are listed in Table S1.

### HCC cell lines

A metastatic HCC cell line, MHCC-97H, was established and provided by the Liver Cancer Institute, Fudan University (Shanghai, China) [31] and cultured following the methods described previously [13]. A metastatic HCC cell line, Huh-7 (JCRB0403), was obtained from the Japanese Cancer Research Resources Bank (JCRB; Osaka, Japan) and cultured following the methods described previously [13]. All cells were cultured at 37 °C in 5% CO_2_.

### Cell transfection assay

For exogenous CXCL11 and LINC00152 overexpression, CAFs were transfected with plasmid-overexpressing CXCL11 or plasmid-overexpressing LINC00152 (CXCL11; LINC00152; GenePharma, Shanghai, China). For LINC00152 knockdown, a vector containing short hairpin RNA for LINC00152 (sh-LINC00152 #1/2; sh-NC as a negative control) was synthesized and obtained from GenePharma. For CXCL11 knockdown, a vector containing short hairpin RNA for CXCL11 (sh-CXCL11) was synthesized and obtained from GenePharma. All transfections were conducted using Lipofectamine 3000 Reagent (Thermo Fisher Scientific, Waltham, MA, USA). The primers used for plasmid construction are listed in Table S1.

### Preparation of conditioned medium (CM)

CM was obtained from CAFs following the methods described previously [13]. Cells and cell debris were removed by passing the collected CM through a 0.2 μm membrane syringe filter.

### Transwell cell migration assay

Transwells without Matrigel gel were used for the migration assays following the methods described previously [13]. Transfected cells were seeded in the upper chambers and DMEM medium containing 10% FBS was added to the lower chambers. At the end of the migration assay, cells that stayed in the upper chambers were discarded, and cells that migrated to the lower chambers were fixed. Next, the cells were stained and counted under an optical microscope.

### Fluorescence in situ hybridization (FISH) assay

A specific Biotin-labeled LINC00152 probe and a DIG-labeled miR-205-5p probe were purchased from General Bio. Ltd. Co, China. After being permeabilized with permeabilizer and digested with proteinase K, cells were prehybridized with a hybridization solution and then incubated with the LINC00152 probe in hybridization buffer overnight at 42 °C. After washing the cells with SSC reagent, they were incubated with HRP-streptavidin for 30 min at 37 °C. Then, the cells were incubated with TSA-520 reagent for 20 min at 37 °C in the dark. Next, the cells were incubated with the miR-205-5p probe, mouse anti-DIG-Biotin and TSA-570 reagent following the above procedures. Cell nuclei were stained with DAPI for 5 min at room temperature. LINC00152 is shown in green florescence and miR-205-5p is shown in red florescence. The cells were imaged by fluorescence microscopy (Olympus).

### Argonaute2 (AGO2) assay

AGO2 was conducted using a Magna RIP™ RNA-binding protein immunoprecipitation kit (Millipore, USA) according to the manufacturer’s guidelines. In short, approximately 1 × 10^7^ cells were lysed and mixed with AGO2 antibody (ab32381, Abcam) or IgG-coated beads on a rotator at 4 °C overnight. Then, the beads were washed, and co-immunoprecipitated RNA was extracted using RNAiso Plus (TaKaRa, Japan), after which the levels of LINC00152, miR-205-5p and the 3’UTR of CXCL11 were detected by RT-qPCR.

### RNA pull-down assay

Biotin-labeled LINC00152 or negative control (NC) oligo probes (General Bio) were pre-incubated with Streptavidin-Dyna beads M-280 (Invitrogen, USA). Next, the cells were crosslinked, lysed and incubated with the beads at 4 °C overnight. Then, the beads were washed, and the crosslinking was reversed. RNA was extracted using RNAiso Plus (TaKaRa, Japan), after which the level of miR-205-5p was measured by RT-qPCR.

### ELISA

Cells, transfected and/or treated, were cultured for 48 h. Then, supernatants were collected, centrifuged (1500 rpm, 5 min), and examined for CXCL11 levels using a human CXCL11 ELISA kit (Invitrogen, USA).

### Immunoblotting assay

Immunoblotting was performed using anti-CXCL11 (CSB-PA06119A0Rb; Cusabio), anti-PCNA (ab29, Abcam, UK) and anti-β-actin (66009-1-Ig; Proteintech, Wuhan, China) antibodies and secondary antibodies (HRP-labeled goat anti-rabbit IgG and goat anti-mouse IgG) following the methods described previously [13] to detect the protein levels of CXCL11 and PCNA. The ECL chemiluminescence method was used to visualize and detect the signals.

### Establishment of a subcutaneous xenotransplanted HCC tumor model in mice

All animal experiments complied with the Guidelines for the Care and Use of Experimental Animals and were approved by the Experimental Committee of the Zhongshan Hospital, Fudan University. All experimental procedures were conducted following the methods described previously [13].

CAFs were infected with CXCL11 overexpression lentivirus (CXCL11) or LINC00152 knockdown lentivirus (sh-LINC00152) for 48 h followed by 4 days of selection with 2 µg/ml puromycin (Beyotime). BALB/c nude mice (SJA Laboratory Animal Co., Ltd, Shanghai, China) were divided into four groups: those recieving MHCC-97H cells + CAFs (NC + sh-NC-infected; n = 6), MHCC-97H cells + CAFs (CXCL11 + sh-NC infected; n = 6), MHCC-97H cells + CAFs (NC + sh-LINC00152 infected; n = 6) and MHCC-97H cells + CAFs (CXCL11 + sh-LINC00152 infected; n = 6). A total of 5 × 10^5^ MHCC-97H cells mixed with 1.5 × 10^6^ infected CAFs were suspended in 100 µl PBS and injected subcutaneously to the left armpit of the mice. After 4 weeks, anesthetized mice were sacrificed, and their tumor volumes and weights were examined. Tumor tissues were collected and subjected to RT-qPCR and immunoblotting.

### Statistics analysis

GraphPad (San Diego, California, USA) was used to process the experimental results and express them as mean ± standard deviation (S.D.). Student’s *t*-test or one-way analysis of variance (ANOVA) followed by Tukey’s multiple comparison test were used to assess statistical significance. *P* value < 0.05 is considered statistically significant.

## Results

### LINC00152 positively regulates CXCL11 levels in CAFs

**Fig. 1:**
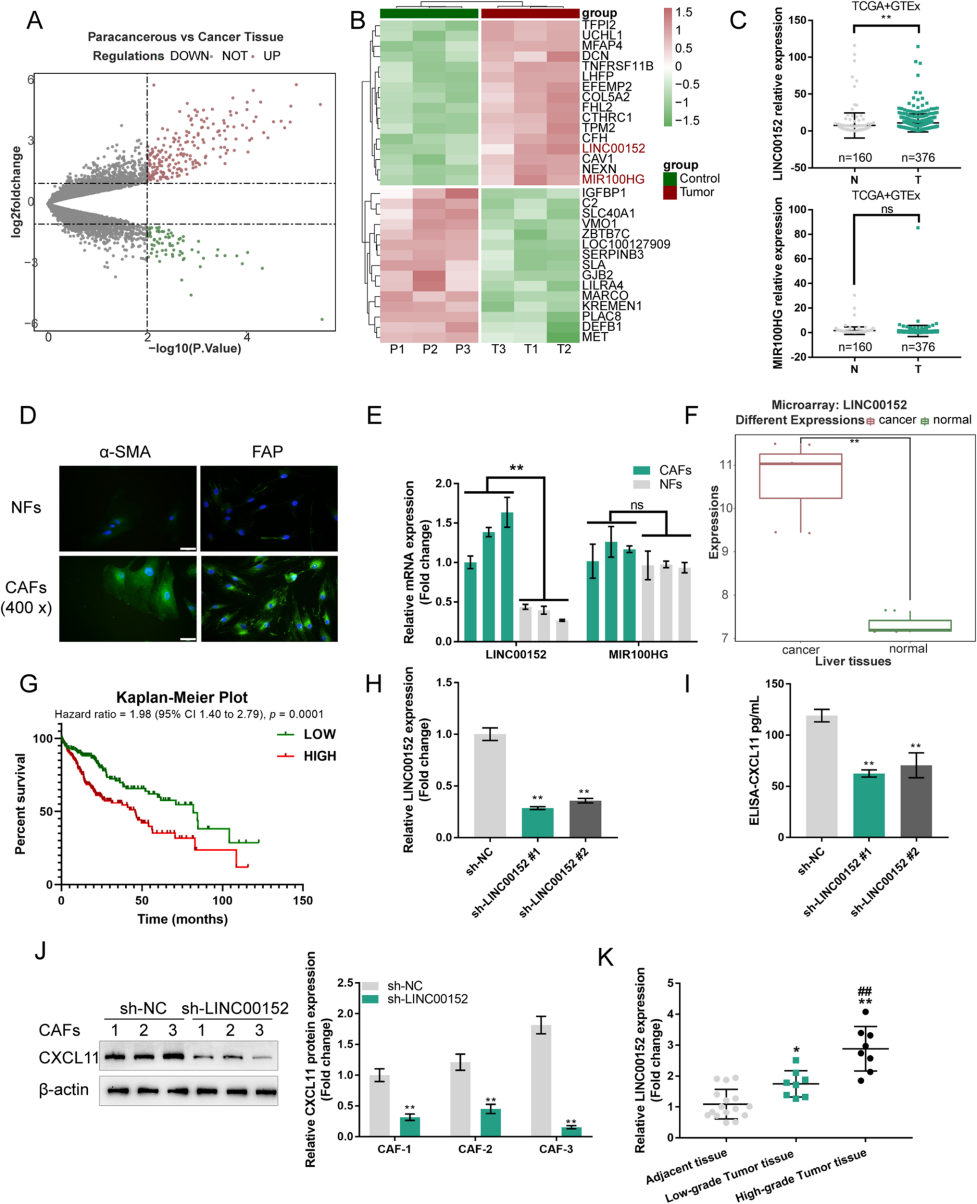
LINC00152 positively regulates CXCL11 levels in CAFs. **A**-**B** Volcano plot and hierarchical clustering heatmap showing differentially expressed lncRNAs in HCC isolated fibroblasts and non-cancer tissue isolated hepatic stellate cells. **C** Expression levels of LINC00152 and MIR100HG in 376 cancer tissues and 160 non-cancer tissues (from TCGA and GTEx). **D** Expression levels of α-SMA and FAP in isolated NFs and CAFs determined by IF staining. **E** Expression levels of LINC00152 and MIR100HG examined in CAFs and NFs by RT-qPCR. **F** LINC00152 expression in HCC tissues and non-cancerous tissues according to microarray data. **G** Kaplan-Meier analysis of the correlation between LINC00152 expression and the survival percentage of HCC patients. **H** LINC00152 knockdown in CAFs by transfecting short hairpin RNAs targeting LINC00152 (sh-LINC00152 #1/2) and LINC00152 confirmed by RT-qPCR. **I** CAFs were transfected with sh-LINC00152 #1/2 after which the levels of CXCL11 in the culture media were examined using ELISA. **J** CAFs were transfected with sh-LINC00152 and examined for CXCL11 protein levels by immunoblotting. **K** Expression levels of LINC00152 in cancer tissues and non-cancer tissues examined by RT-qPCR. * *p* < 0.05, ** *p* < 0.01 compared to normal adjacent tissues, NFs or sh-NC group. ^##^ *p* < 0.01 compared to low-grade tumor tissues

As reported previously, neutralizing CAF-secreted CXCL11 can partially attenuate HCC cell proliferation and migration. Thus, we searched for factors that might modulate CXCL11 expression in CAFs. By comparing differentially expressed lncRNAs in fibroblasts and hepatic stellate cells (HSCs) from HCC cancer and non-cancer tissue samples, respectively, according to our previously reported microarray chip assay [32], we found 72 down-regulated and 211 up-regulated genes (logFC > 1 or < -1, *p* < 0.01) in HCC isolated fibroblasts (Fig. 1A), the top 31 differently expressed genes are shown in Fig. 1B. Among those differently expessed genes, only LINC00152 and MIR100HG represented non-coding RNAs. The expression of LINC00152 was significantly up-regulated (logFC = 3.368, P = 3.4e-03) in HCC-associated fibroblasts (Fig. 1A-B). To further select the most critical lncRNA, LINC00152 and MIR100HG expression in 376 cancer tissue samples and 160 non-cancer tissue samples from TCGA and GTEx was determined. We found that only LINC00152 expression was dramatically increased in cancer tissue samples (Fig. 1C). Next, isolated NFs and CAFs were identified by IF staining of α-SMA and FAP (Fig. 1D). The CAFs showed higher levels of α-SMA and FAP. Next, LINC00152 and MIR100HG expression was examined in CAFs and NFs. We found that only LINC00152 expression was significantly increased in CAFs (Fig. 1E). According to our microarray data, the expression level of LINC00152 showed a marked up-regulation in HCC tissue samples compared to that in non-cancer adjacent tissue samples (Fig. 1F). Moreover, based on Kaplan-Meier analysis, a higher expression of LINC00152 was found to predict a lower survival percentage of HCC patients (Fig. 1G).

To investigate regulation of CXCL11 by LINC00152, we achieved LINC00152 knockdown in CAFs by transfecting a short hairpin RNA targeting LINC00152 (sh-LINC00152 #1/2). LINC00152 knockdown was confirmed by RT-qPCR (Fig. 1H). In CAFs transfected with sh-LINC00152 #1/2, the levels of CXCL11 in the culture medium were found to be dramatically decreased compared with those of CAFs transfected with sh-NC (Fig. 1I). In CAFs transfected with sh-LINC00152, the protein levels of CXCL11 were significantly decreased compared to those in CAFs transfected with sh-NC (Fig. 1J). Markedly, LINC00152 expression was found to be increased in HCC tumor tissue samples compared to non-cancer adjacent tissue samples (Fig. 1K). Thus, LINC00152 may regulate CXCL11 expression in CAFs.

### Co-effects of LINC00152 and CXCL11 in CAFs on HCC cells

**Fig. 2:**
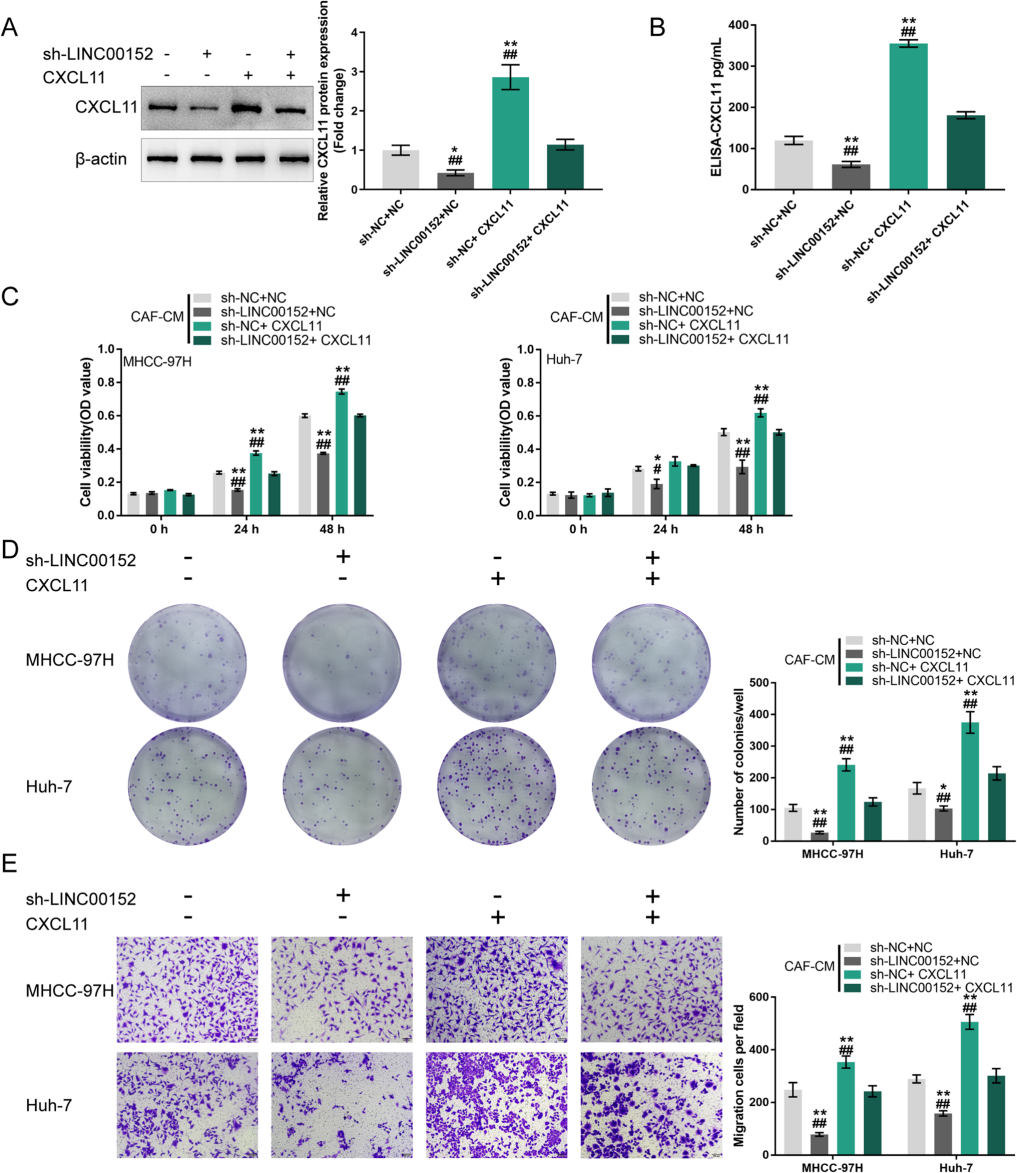
Co-effects of LINC00152 knockdown and CXCL11 overexpression in CAFs on HCC cells. **A**-**B** CAFs were co-transfected with sh-LINC00152 and CXCL11 and examined for CXCL11 protein levels by immunoblotting and CXCL11 protein levels in culture media by ELISA. Then, CAFs were co-transfected with sh-LINC00152/sh-NC and CXCL11/NC vector, after which the collected conditioned culture media (CM) were used for HCC cell culture. **C** HCC cell viability determined by MTT assay. **D** HCC cell colony forming ability determined by colony formation assay. **E** HCC cell migration determined by Transwell assay. * *p* < 0.05, ** *p* < 0.01 vs. sh-NC + NC group;^#^ *p* < 0.05, ^##^ *p* < 0.01 vs. sh-LINC00152 + CXCL11 group

**Fig. 3:**
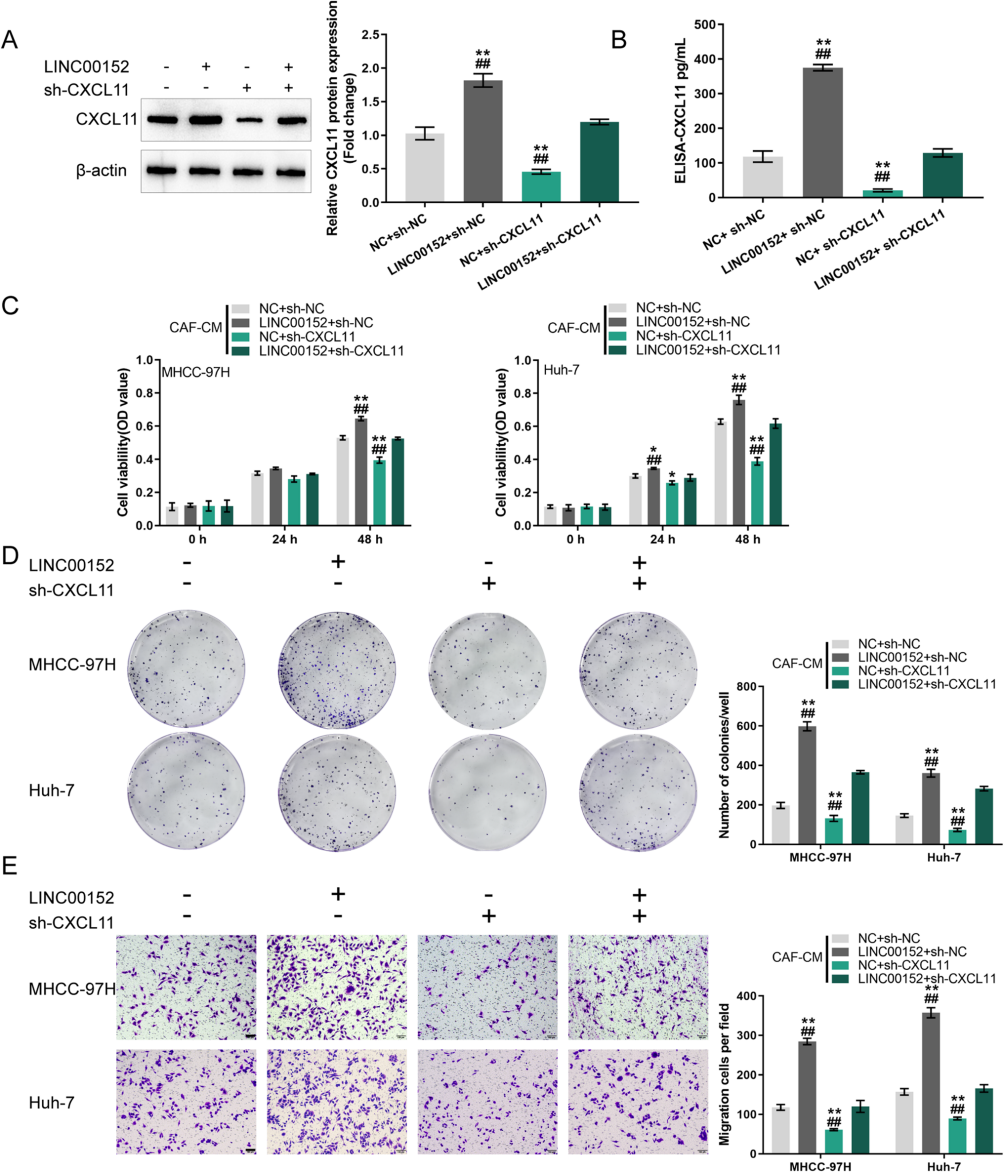
Co-effects of LINC00152 overexpression and CXCL11 knockdown in CAFs on HCC cells. **A**-**B** CAFs were co-transfected with LINC00152 overexpression vector and sh-CXCL11 and examined for CXCL11 protein levels by immunoblotting and CXCL11 protein levels in culture media by ELISA. Then, CAFs were co-transfected with LINC00152/NC and sh-CXCL11/sh-NC vector, after which collected conditioned culture media (CM) were used for HCC cell culture. **C** HCC cell viability determined by MTT assay. **D** HCC cell colony forming ability determined by colony formation assay. **E** HCC cell migration determined by Transwell assay. ** *p* < 0.01 vs. sh-NC + NC group; ^##^ *p* < 0.01 vs. LINC00152 + sh-CXCL11 group

To validate the co-effects of LINC00152 and CXCL11 in CAFs on HCC cells, we co-transfected CAFs with sh-LINC00152 and CXCL11 and examined CXCL11 protein levels in CAFs and culture media. We found that LINC00152 knockdown decreased CXCL11 levels in CAFs and culture media, whereas CXCL11 overexpression in CAFs attenuated the inhibitory effects of sh-LINC00152 on CXCL11 levels (Fig. 2A-B). Then, CAFs were co-transfected with sh-LINC00152/sh-NC and CXCL11/vector, after which the conditioned media (CM) were used for HCC cell culture. We found that CAF-CM (sh-LINC00152) considerably suppressed HCC cell viability, colony formation and migration, whereas CAF-CM (CXCL11) exerted opposite effects. The effects of LINC00152 knockdown in CAFs could be partially attenuated by CXCL11 overexpression (Fig. 2C-E). In contrast, we found that LINC00152 overexpressing CAFs exhibited higher CXCL11 levels in cells and culture media. CXCL11 knockdown reduced LINC00152-induced upregulation of CXCL11 (Fig. 3A-B). Next, we found that CAF-CM (LINC00152) considerably increased HCC cell viability, colony formation and migration, whereas CAF-CM (sh-CXCL11) exerted opposite effects. The effects of LINC00152 overexpression in CAFs could be partially attenuated by CXCL11 knockdown (Fig. 3C-E). These data indicate that cross-talk between LINC00152 and CXCL11 in CAFs affects HCC cell phenotypes.

### Co-effects of LINC00152 knockdown and CXCL11 overexpression in CAFs on tumor growth in a mouse model

**Fig. 4:**
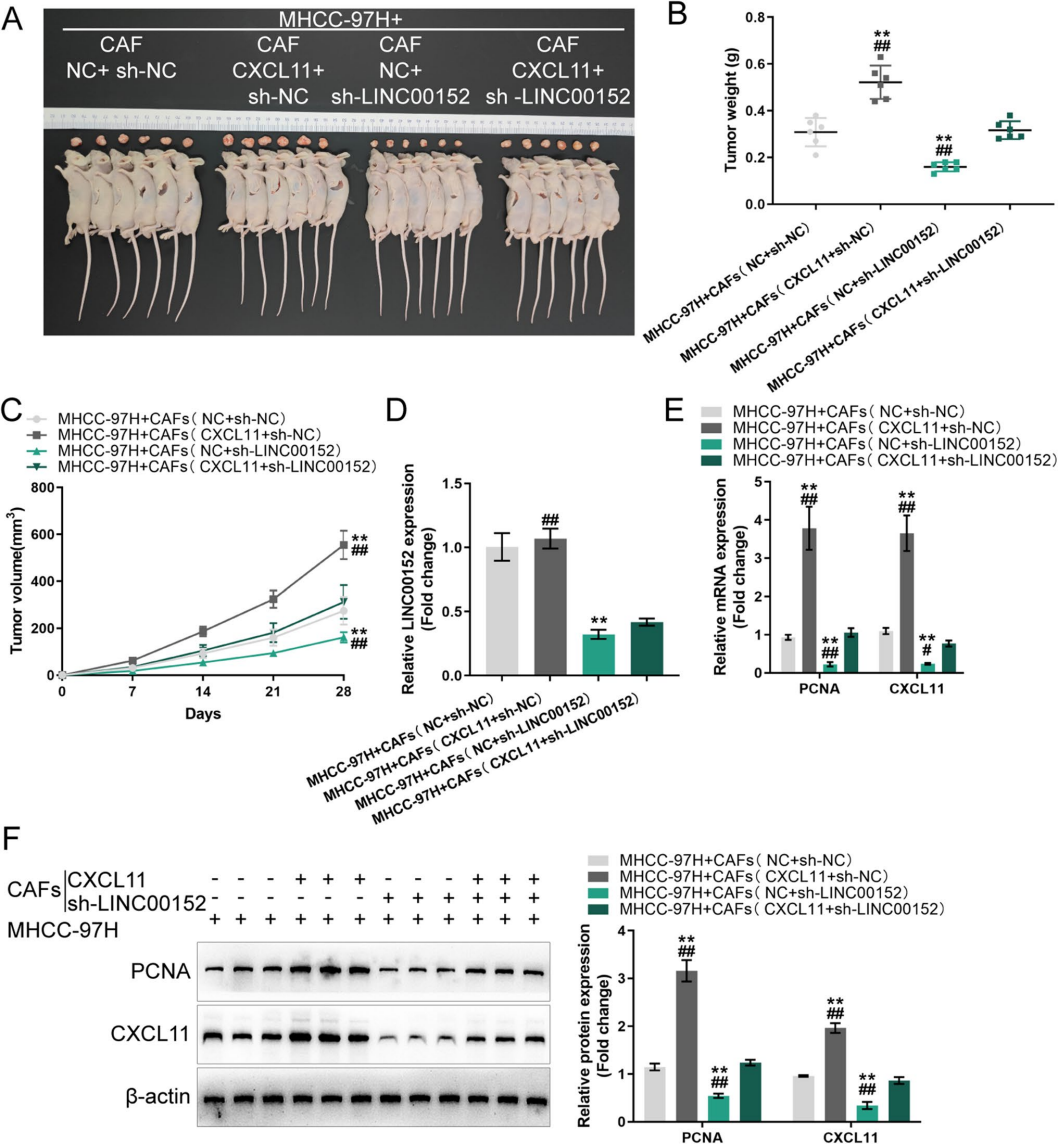
LINC00152 knockdown and CXCL11 overexpression in CAFs modulates tumor growth in a mouse model. **A** Mice were divided into four groups: those recieving MHCC-97H cells + CAFs (NC + sh-NC infected; n = 6), MHCC-97H cells + CAFs (CXCL11 + sh-NC infected; n = 6), MHCC-97H cells + CAFs (NC + sh-LINC00152 infected; n = 6) and MHCC-97H cells + CAFs (CXCL11 + sh-LINC00152 infected; n = 6). A total of 5 × 10^5^ MHCC-97H cells mixed with 1.5 × 10^6^ transfected CAFs were suspended in 100 µl PBS and subcutaneously injected into the left armpit of nude mice. **B**-**C** After 28 days, anesthetized mice were sacrificed and the tumor volumes and weights were examined. **D** Expression level of LINC00152 in tumor tissues determined by RT-qPCR. **E**-**F** Tumor tissues were collected after which the mRNA and protein expression levels of PCNA and CXCL11 in tumor tissues were examined using RT-qPCR and immunoblotting, respectively. ** *p* < 0.01 vs. MHCC-97H + CAFs (NC + sh-NC) group;^#^ *p* < 0.05, ^##^ *p* < 0.01 vs. MHCC-97H + CAFs (CXCL11 + sh-LINC00152) group

After establishing a subcutaneous xenotransplanted tumor model of CAFs plus HCC cells in mice, we set out to validate the effects of LINC00152 knockdown and CXCL11 overexpression on tumor growth *in vivo*. The mice were divided into four groups: those recieving MHCC-97H cells + CAFs (NC + sh-NC-infected; n = 6), MHCC-97H cells + CAFs (CXCL11 + sh-NC-infected; n = 6), MHCC-97H cells + CAFs (NC + sh-LINC00152-infected; n = 6) and MHCC-97H cells + CAFs (CXCL11 + sh-LINC00152-infected; n = 6). MHCC-97H cells mixed with lentivirus infected CAFs (tumor cells:NFs or CAFs = 1:3) were subcutaneously injected into the left armpits of nude mice and allowed to grow for 28 days (Fig. 4A). Next, the tumor volumes and weights were examined (Fig. 4B-C). We found that the tumor volumes and weights of the MHCC-97H cells + CAFs (CXCL11 + sh-NC) were higher, and those of the MHCC-97H cells + CAFs (NC + sh-LINC00152) group were lower than those of the MHCC-97H cells + CAFs (NC + sh-NC) group. The tumor volumes and weights of the MHCC-97H cells + CAFs (CXCL11 + sh-LINC00152) group were similar to those of the MHCC-97H cells + CAFs (NC + sh-NC) group. Within the tumor tissues, the level of LINC00152 decreased in the MHCC-97H + CAFs (NC + sh-LINC00152) / (CXCL11 + sh-LINC00152) groups (Fig. 4D). PCNA and CXCL11 mRNA and protein expression was found to be dramatically higher in the MHCC-97H cells + CAFs (CXCL11 + sh-NC) group, and partially downregulated in the MHCC-97H cells + CAFs (NC + sh-LINC00152) group compared to the MHCC-97H cells + CAFs (NC + sh-NC) group (Fig. 4E-F). No significant difference was observed between the MHCC-97H cells + CAFs (NC + sh-NC) group and MHCC-97H cells + CAFs (CXCL11 + sh-LINC00152) group. Thus, LINC00152 knockdown in CAFs can reverse CXCL11 overexpression in CAF-induced tumor growth in a mouse model.

### miR-205-5p directly targets LINC00152 and the 3’UTR of CXCL11

**Fig. 5:**
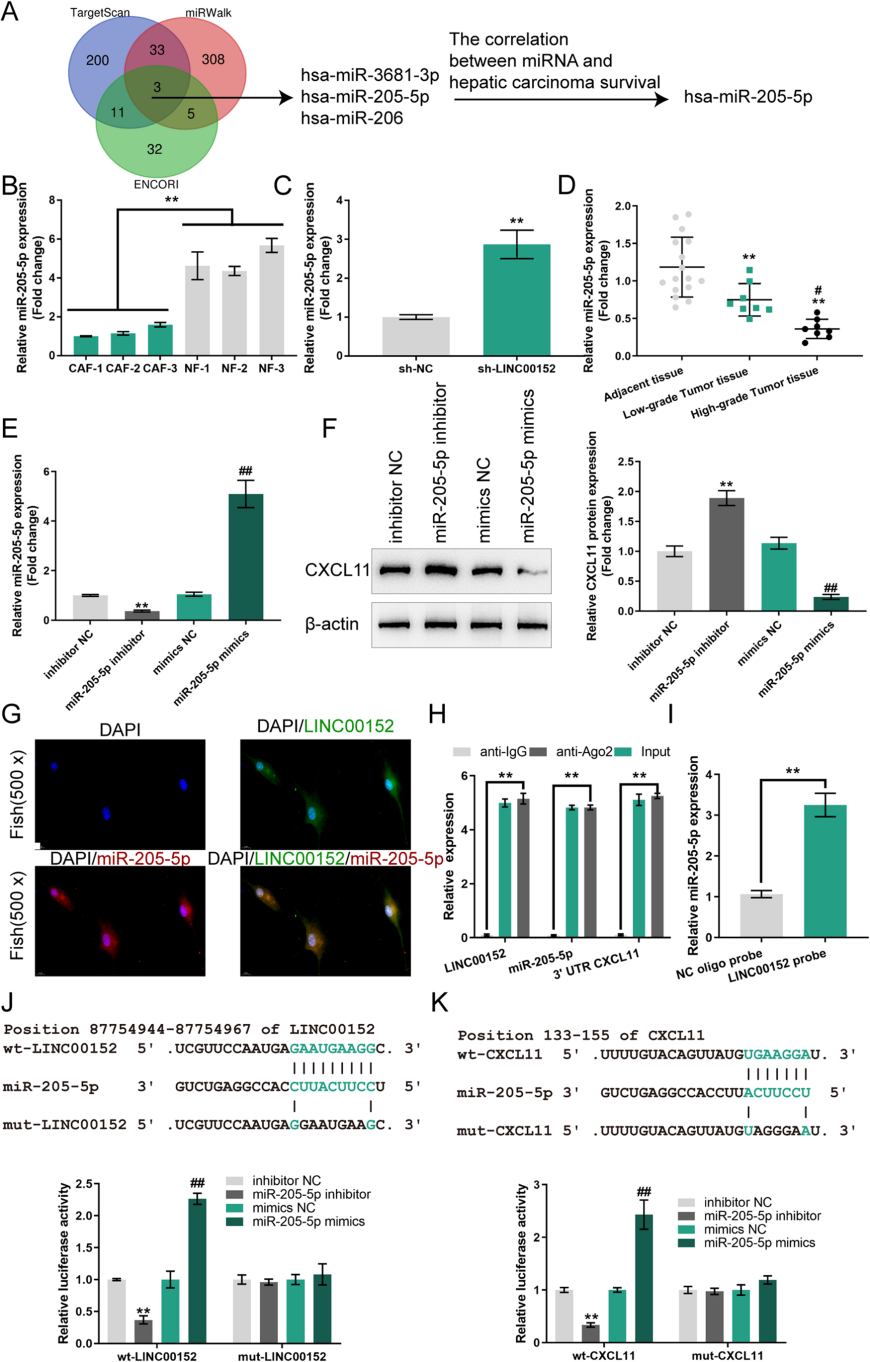
miR-205-5p directly targets LINC00152 and CXCL11. **A** Targetscan, miRWalk and ENCORI were used to predict miRNAs that might target LINC00152 and CXCL11; three miRNAs were obtained. The correlation between these miRNAs and HCC patient survival was analyzed, after which miR-205-5p was selected. **B** Expression of miR-205-5p examined in CAFs and NFs using RT-qPCR. (C) CAFs were transfected with sh-NC or sh-LINC00152 and examined for miR-205-5p expression using RT-qPCR. **D** miR-205-5p expression examined in HCC and non-cancer adjacent tissues using RT-qPCR. **E** miR-205-5p overexpression or inhibition was achieved in CAFs by transfecting miR-205-5p mimics or inhibitor. miR-205-5p overexpression or inhibition was confirmed using RT-qPCR. **F** CAFs were transfected with miR-205-5p mimics or inhibitor and examined for CXCL11 protein levels by immunoblotting. **G** Co-location of LINC00152 and miR-205-5p determined by FISH assay. **H** AGO2 assay. **I** Biotinylated RNA-pulldown assay. **J**-**K** Wild-type and mutant-type LINC00152 or CXCL11 luciferase reporter vectors were constructed and co-transfected into 293T cells with miR-205-5p mimics or inhibitor; luciferase activity was determined. ** *p* < 0.01 vs.sh-NC or inhibitor NC group; ^##^ *p* < 0.01 vs. mimics NC group

miRNAs have been reported to mediate cross-talk between lncRNAs and mRNAs [33, 34]. Therefore, we next used Targetscan, miRWalk and ENCORI to predict miRNAs that might target LINC00152 and CXCL11. Three miRNAs were obtained: miR-3681-3p, miR-205-5p and miR-206 (Fig. 5A). Correlation between these miRNAs and HCC patient survival was analyzed, after which miR-205-5p was selected because its expression correlated with overall survival (Fig. S1). In contrast to LINC00152 and CXCL11, miR-205-5p expression was dramatically downregulated in CAFs compared to that in NFs (Fig. 5B). In CAFs transfected with sh-LINC00152, the expression level of miR-205-5p was found to be markedly increased (Fig. 5C). Consistently, the expression level of miR-205-5p was found to be dramatically downregulated in HCC tissue samples compared to that in non-cancer adjacent tissue samples (Fig. 5D).

To further investigate the predicted regulation, miR-205-5p overexpression/inhibition in CAFs was achieved by transfecting miR-205-5p mimics/inhibitor. miR-205-5p overexpression/inhibition was confirmed using RT-qPCR (Fig. 5E). We found that in the CAFs miR-205-5p overexpression reduced, while miR-205-5p inhibition elevated CXCL11 protein levels (Fig. 5F). In addition, the reported target genes of miR-205-5p, SEMA4C and PLCβ1, were set as positive controls. We found that they were also regulated by miR-205-5p (Fig. S2). Using FISH, we found that LINC00152 and miR-205-5p co-located both in the nucleus and the cytoplasm (Fig. 5G). AGO2 assays further confirmed that LINC00152, miR-205-5p and the 3’UTR of CXCL11 were enriched after anti-AGO2 immunoprecipitation compared with IgG (Fig. 5H). RNA-pulldown assays with a biotin-LINC00152 probe were subsequently performed to confirm that miR-205-5p was enriched in LINC00152 probed RNA-RNA complexes using RT-qPCR (Fig. 5I). Next, we constructed wild-type and mutant-type LINC00152 or 3’UTR CXCL11 luciferase reporter vectors, co-transfected these vectors into 293T cells with miR-205-5p mimics/inhibitor, and examined luciferase activity. When co-transfected with wild-type LINC00152 or CXCL11 luciferase reporter vectors, miR-205-5p overexpression inhibited, whereas miR-205-5p inhibition enhanced the luciferase activity (Fig. 5J-K). When co-transfected with mutant-type LINC00152 or CXCL11 luciferase reporter vectors, neither miR-205-5p overexpression nor miR-205-5p inhibition altered the luciferase activity (Fig. 5J-K). From these results we conclude that miR-205-5p directly targets LINC00152 and CXCL11.

### Co-effects of LINC00152 and miR-205-5p upon CXCL11 production in CAFs and HCC cells

**Fig. 6:**
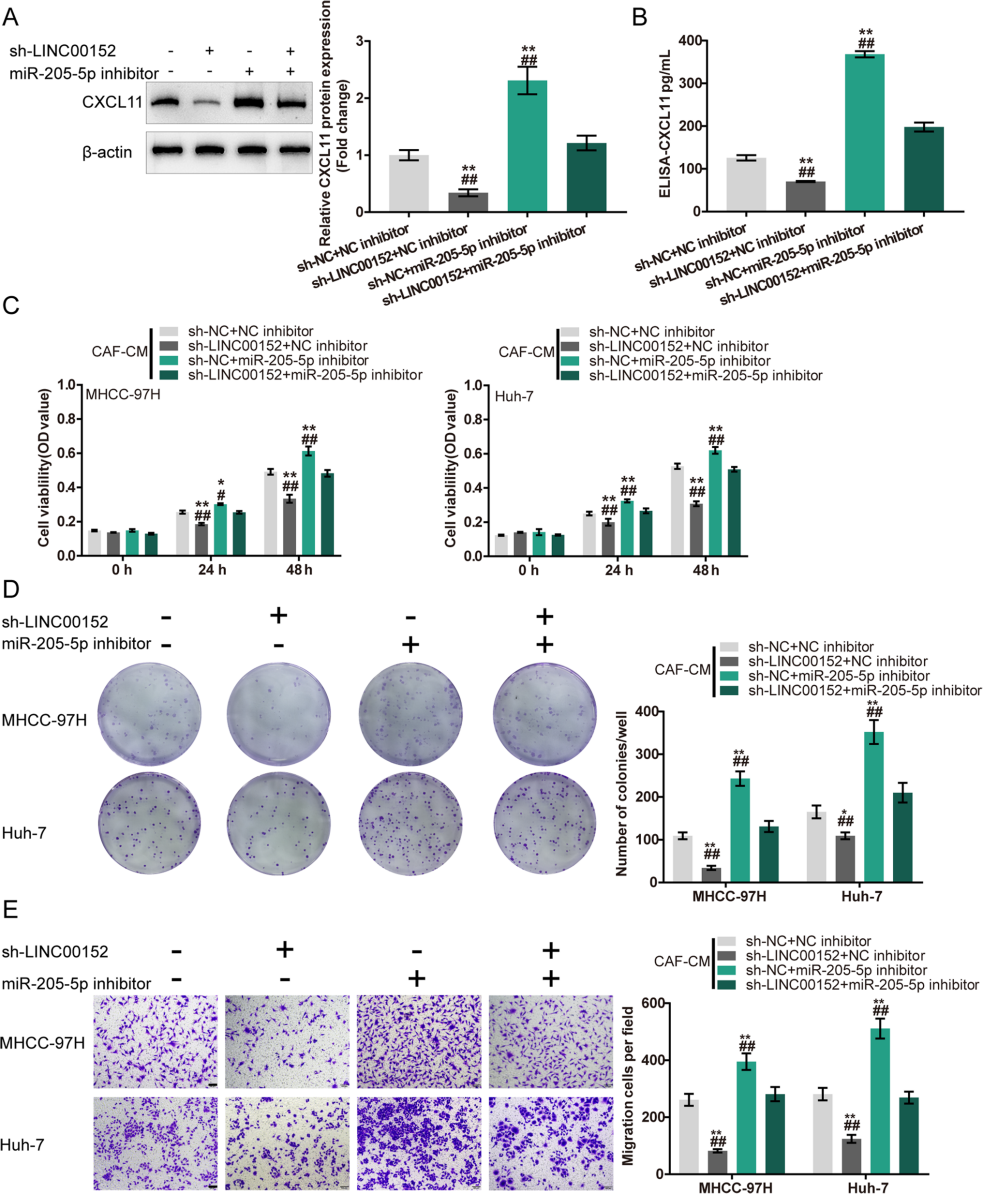
Co-effects of LINC00152 knockdown and miR-205-5p inhibitor in CAFs on CXCL11 production and HCC cells. CAFs were co-transfected with sh-LINC00152 and miR-205-5p inhibitor and examined for CXCL11 protein levels by immunoblotting (**A**) and CXCL11 protein levels in culture media by ELISA (**B**). Then, the conditioned media were obtained from CAFs co-transfected with sh-LINC00152/sh-NC and miR-205-5p inhibitor/NC inhibitor, after which HCC cells were cultured with collected conditioned media (CM) and examined for viability by MTT assay (**C**), colony forming ability by colony formation assay (**D**) and cell migration by Transwell assay (E). * *p* < 0.05, ** *p* < 0.01 vs. sh-NC + NC inhibitor group; ^#^ *p* < 0.05, ^##^ *p* < 0.01 vs. sh-LINC00152 + miR-205-5p inhibitor

**Fig. 7:**
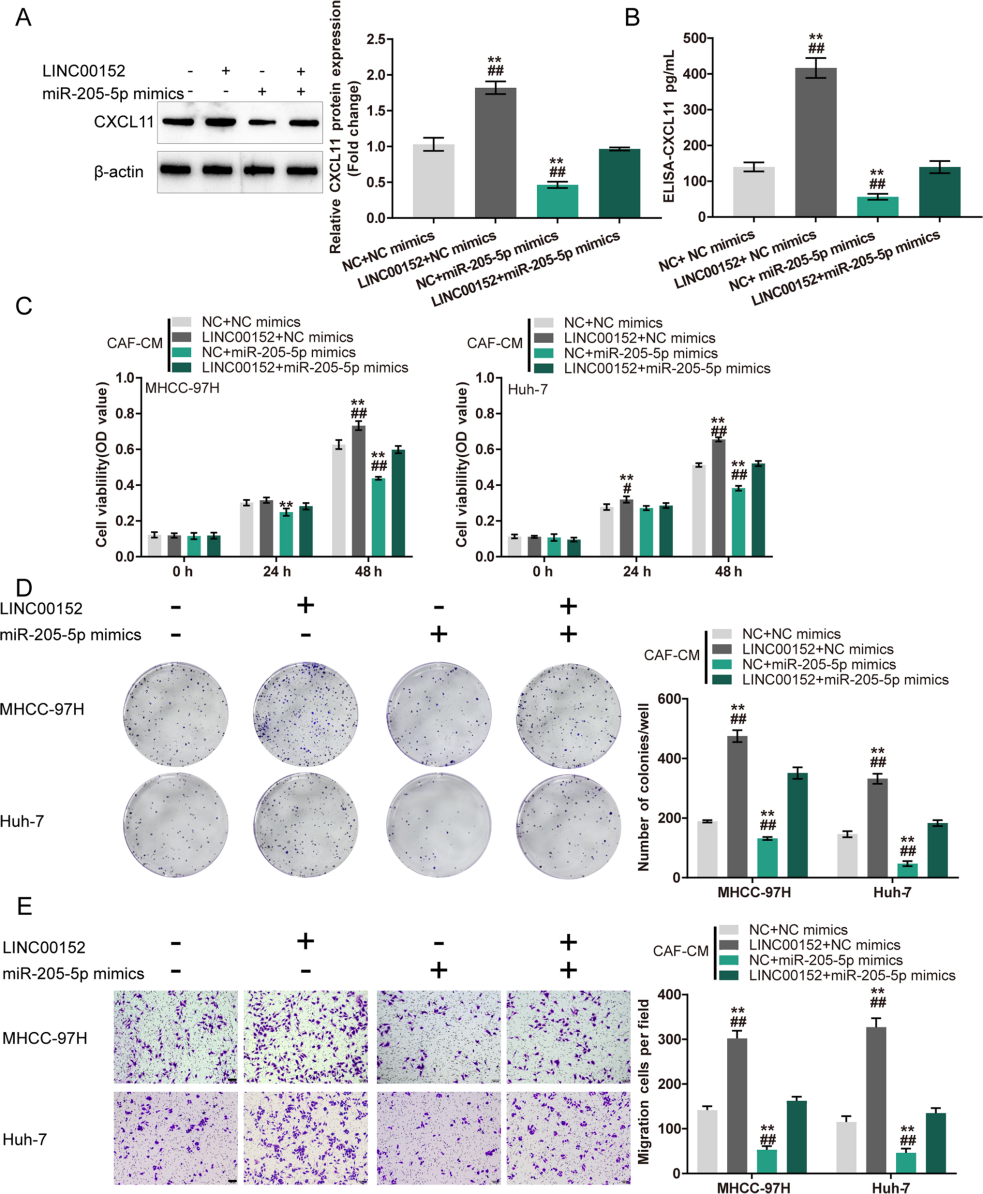
Co-effects of LINC00152 overexpression and miR-205-5p mimics upon CXCL11 production in CAFs and HCC cells. CAFs were co-transfected with LINC00152 and miR-205-5p mimics and examined for CXCL11 protein levels by immunoblotting (**A**) and CXCL11 protein levels in culture media by ELISA (**B**). Then, conditioned media were obtained from CAFs co-transfected with LINC00152/NC and miR-205-5p mimics/NC mimics, after which HCC cells were cultured with collected conditioned media (CM) and examined for cell viability by MTT assay (**C**), colony forming ability by colony formation assay (**D**) and cell migration by Transwell assay (**E**). ** *p* < 0.01 vs. NC + NC mimics group; ^##^ *p* < 0.01 vs. LINC00152 + miR-205-5p mimics

After confirming miR-205-5p binding to LINC00152 and CXCL11, we set out to investigate the co-effects of the LINC00152/miR-205-5p axis in CAFs on HCC cell phenotypes. We co-transfected CAFs with sh-LINC00152 and miR-205-5p inhibitor and determined CXCL11 protein contents in CAFs and culture media. We found that LINC00152 knockdown decreased, whereas miR-205-5p inhibition increased the CXCL11 protein contents in CAFs and culture media. miR-205-5p inhibition significantly attenuated the effects of LINC00152 knockdown (Fig. 6A-B). Then, conditioned media (CMs) were obtained from CAFs co-transfected with sh-LINC00152/sh-NC and miR-205-5p inhibitor/NC inhibitor. HCC cells were cultured with the CMs and examined phenotypically. We found that CAF-CM (sh-LINC00152) inhibited HCC cell viability, colony formation and migration, whereas CAF-CM (miR-205-5p inhibitor) exerted opposite effects on HCC cell phenotypes. The effects induced by LINC00152 knockdown were partially reversed by miR-205-5p inhibition (Fig. 6C-E). In contrast, CAFs with LINC00152 overexpression exhibited higher CXCL11 levels both within cells and culture media. miR-205-5p mimics reduced LINC00152-induced upregulation of CXCL11 (Fig. 7A-B). In addition, we found that the CAF-CM (LINC00152 + NC mimics) considerably increased HCC cell viability, colony formation and migration, whereas CAF-CM (NC + miR-205-5p mimics) exerted opposite effects. miR-205-5p205-5p mimics in CAFs could partially attenuate the effects of LINC00152 overexpression (Fig. 7C-E). These data indicate that cross-talk between LINC00152 and miR-205-5p in CAFs affects HCC cell phenotypes.

## Discussion

Here, we found that CAF-secreted CXCL11 promotes HCC cell proliferation and migration through the LINC00152/miR-205-5p/CXCL11 axis. *In vivo*, CM from CAFs transfected with sh-LINC00152 or sh-CXCL11 dramatically suppressed HCC cell viability, colony formation and migration, whereas CAF-CM (CXCL11 or LINC00152 overexpression) exerted opposite effects. The effects of LINC00152 knockdown in CAFs could be partially attenuated by CXCL11 overexpression. In vivo, LINC00152 knockdown in CAFs inhibited HCC tumor growth in a mouse model, which could be reversed by CXCL11 overexpression in CAFs. We found that miR-205-5p directly binds to LINC00152 and the 3’UTR of CXCL11 and that through this binding, LINC00152 served as a ceRNA to counteract miR-205-5p-mediated suppression on CXCL11 in CAFs, which affected HCC cell phenotypes.

As mentioned above, lncRNAs are known to play key roles in regulating the liver microenvironment and chronic hepatic disorders through immune reaction, hepatic regeneration and redox signaling pathways. Dysregulation of lncRNAs within these processes results in chronic hepatitis, hepatic outgrowth and oxidative stress, leading to HCC development and progression [35]. According to previous studies, CAFs can modulate the expression of pro-inflammatory genes to maintain a liver inflammatory microenvironment [4, 9] and, consequently, enhance HCC progression and metastasis. By analyzing microarray chip data, we observed LINC00152 upregulation in HCC and CAFs. In CAFs, LINC00152 knockdown decreased CXCL11 levels in both the cells and culture media, suggesting that LINC00152 in CAFs may be related to its effects on HCC cells. LINC00152 has been reported to be upregulated in gastric cancer [36, 37], colon cancer [38], gallbladder cancer [39], renal cell carcinoma [40] and HCC [41]. Notably, LINC00152 has emerged as a key oncogenic lncRNA in various types of malignant tumors [42]. Therefore, LINC00152 may also exert oncogenic functions during HCC development. As expected, LINC00152 knockdown in CAFs decreased CXCL11 levels in both the cells and culture media, subsequently inhibiting HCC cell proliferation and migration. Besides, co-injection with CAFs transfected with sh-LINC00152 slowed down the growth of tumors in mouse models, confirming that knocking down LINC00152 in CAFs is tumor inhibitory.

LncRNAs can play their roles via various mechanisms. Currently, however, the ceRNA hypothesis is attracting more attention because miRNAs can mediate cross-talk between lncRNAs and mRNAs. Here, we searched for miRNAs that showed positive correlations with LINC00152 and CXCL11 in CAFs and identified miR-205-5p. Through binding, we found that miR-205-5p inhibited the expression of CXCL11, and that LINC00152 counteracted miR-205-5p-mediated suppression of CXCL11. As recently reported, miR-205-5p can exert oncogenic or tumor-suppressive effects on various tumor cells [14–18]. In HCC, Zhang et al. [43] demonstrated that exogenous miR-205-5p expression significantly suppressed HBX-enhanced HCC cell proliferation both in vitro and *in vivo*, indicating that miR-205-5p has the potential to act as a tumor suppressor during HCC development. Shao et al. [44] revealed that miR-205-5p can target the PTEN/JNK/ANXA3 pathway to regulate the chemoresistance of HCC cells. Lu et al. found that miR-205-5p can bind SEMA4C to suppress HCC tumor growth, invasion and EMT [45]. Moreover, downregulation of miR-205-5p promoted the stemness of HCC cells via targeting PLCβ1 [46]. These studies only investigated the direct effects of miR-205-5p on HCC cells. In this study, we found that miR-205-5p inhibition in CAFs increased CXCL11 levels in both the cells and culture media, thus promoting the capacity of HCC cells to proliferate and migrate. More importantly, miR-205-5p inhibition in CAFs could significantly attenuate the effects of LINC00152 silencing in CAFs, suggesting that the LINC00152/miR-205-5p axis in CAFs can modulate the expression of CXCL11 and, consequently, affect HCC cell phenotypes. miR-205-5p has been reported to modulate the expression of other cytokines in different cancer types. In oral cancer cells, miR-205-5p has been found to promote IL-24 expression [47], whereas in melanoma cells, miR-205-5p has been found to inhibit CCL18 release via targeting its 3’UTR [48]. Therefore, it needs to be further investigated whether in CAFs miR-205-5p modulates these cytokines to affect the HCC phenotypes.

Together, our data indicate that a LINC00152/miR-205-5p/CXCL11 axis in HCC CAFs may affect the proliferation and migration of HCC cells in vitro and HCC tumor growth *in vivo*.

## Supplementary Information

Fig. 1 The correlation between miRNAs expression (miR-3681, miR-205 and miR-206) and HCC patients’ survival was analyzed. (JPG 572 KB)

Fig. 2 The expression of miR-205-5p reported target genes. (PNG 174 kb)

High Resolution Image (TIF 869 kb)

Table S1 (DOCX 18 kb)

## Data Availability

All data generated or analysed during this study are included in this published article (and its supplementary information files).
