# Supplementary figures and images for: The LINC00152/miR-205-5p/CXCL11 axis in hepatocellular carcinoma cancer-associated fibroblasts affects cancer cell phenotypes and tumor growth
Source: Cell Oncol (Dordr). 2022 Nov 26;45(6):1435–49. doi: 10.1007/s13402-022-00730-4 (PMC9747837; doi:10.1007/s13402-022-00730-4)

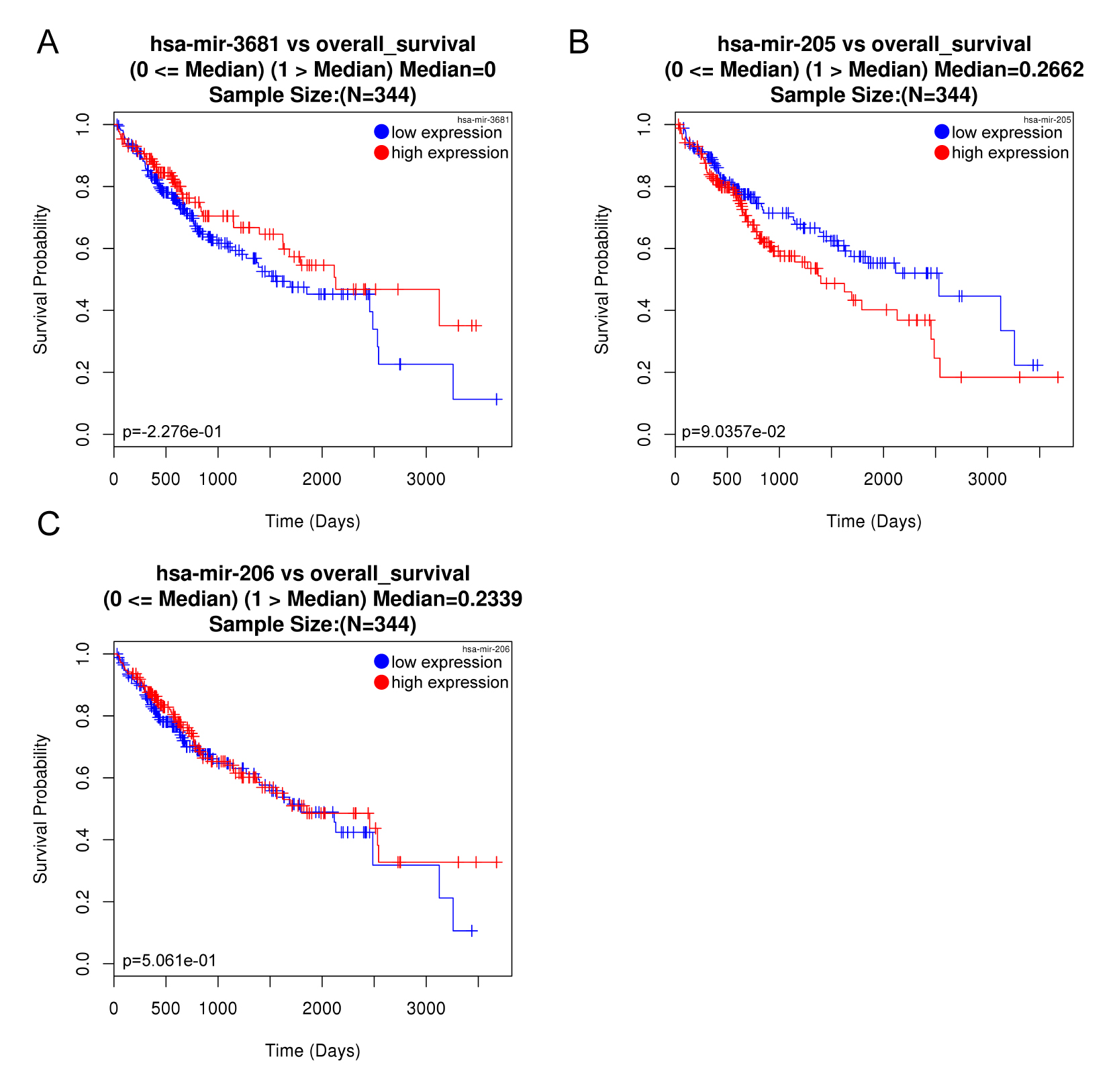

Supplement: Supplementary file 1 — The correlation between miRNAs expression (miR-3681, miR-205 and miR-206) and HCC patients’ survival was analyzed. (JPG 572 KB) [file 13402_2022_730_MOESM1_ESM.jpg]

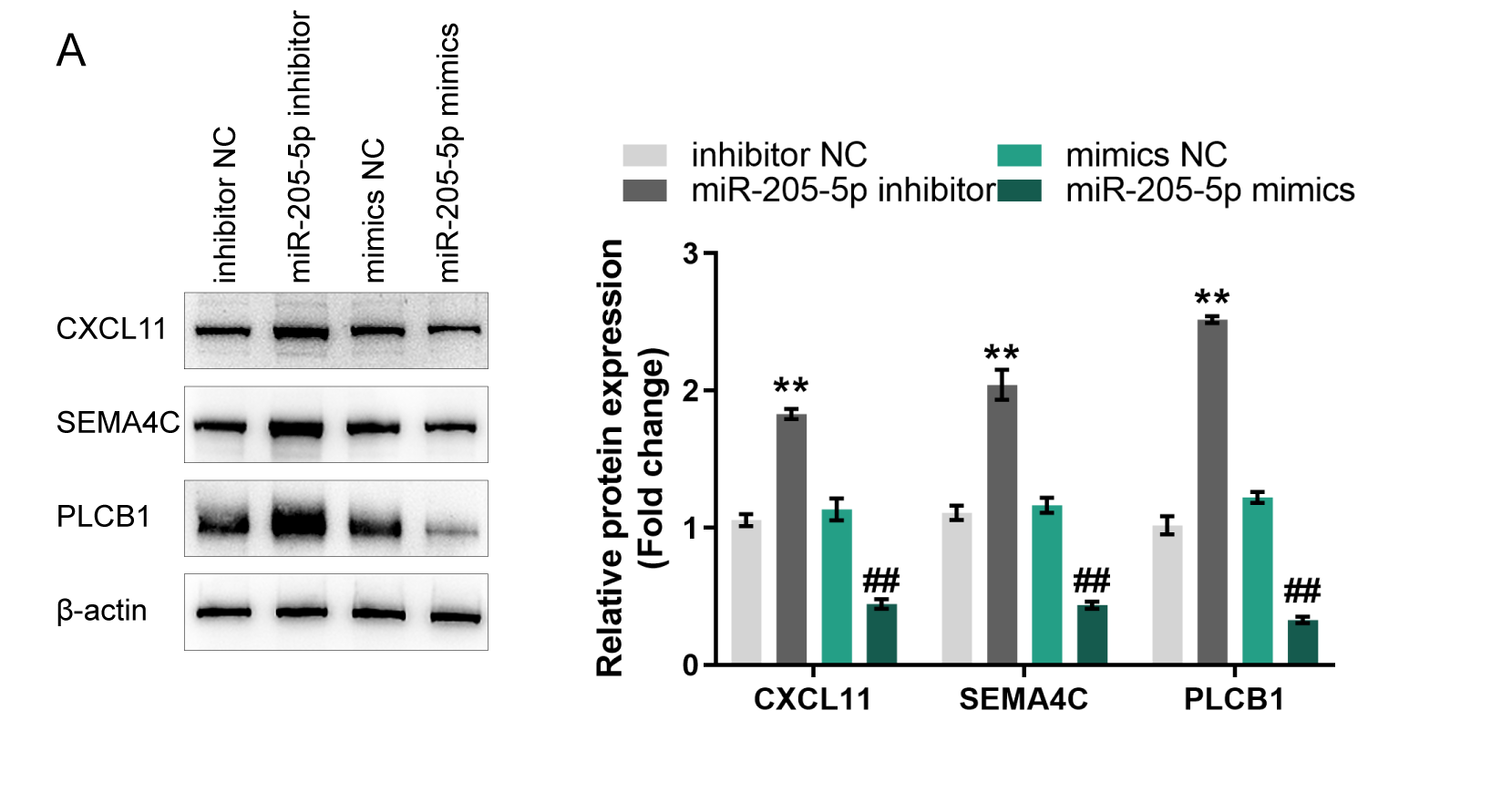

Supplement: Supplementary file 2 — The expression of miR-205-5p reported target genes. (PNG 174 kb) [file 13402_2022_730_Fig8_ESM.png]

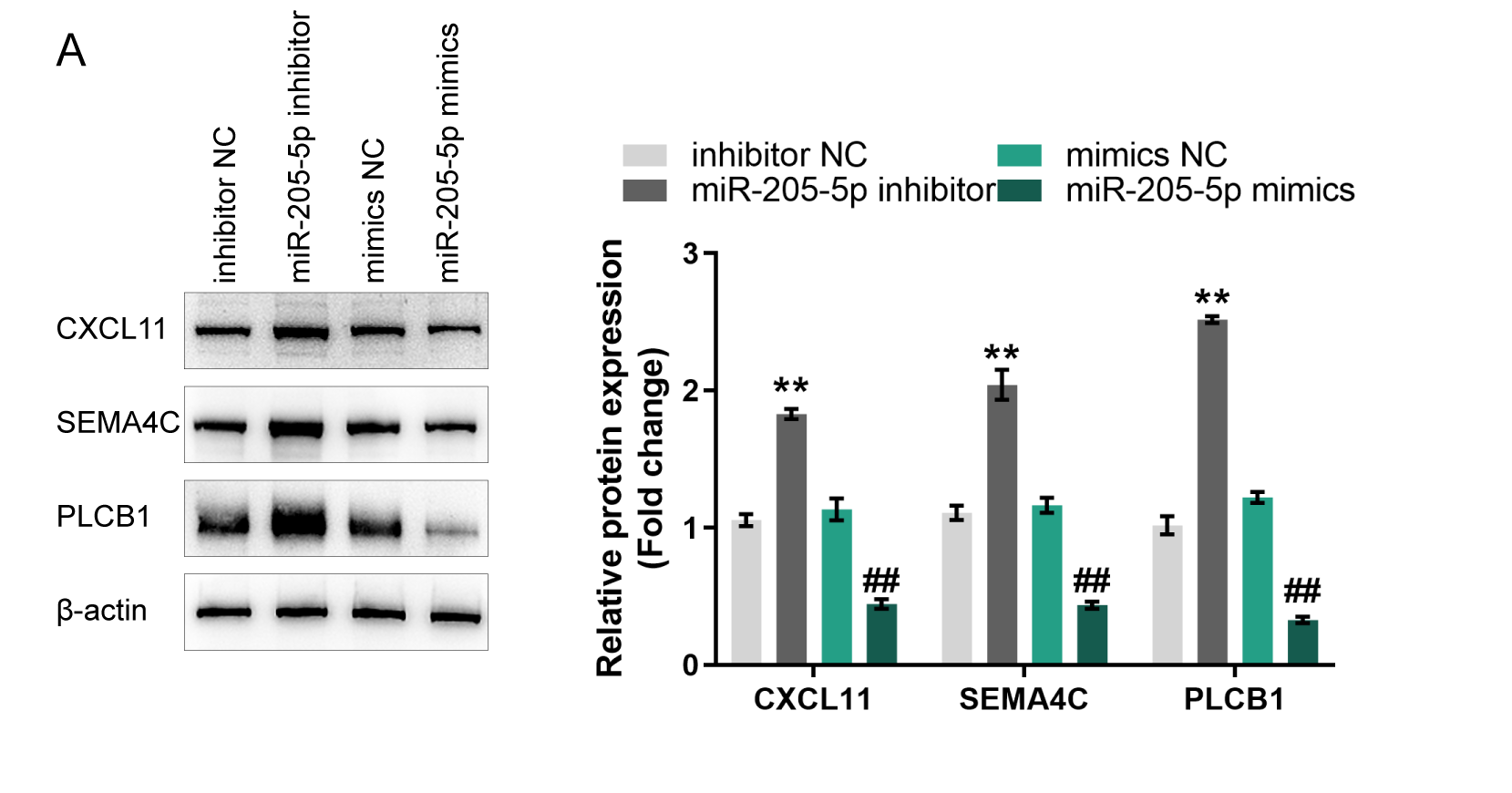

Supplement: Supplementary file 3 — High Resolution Image (TIF 869 kb) [file 13402_2022_730_MOESM2_ESM.tif]
