# Supplementary material for: The LINC00152/miR-205-5p/CXCL11 axis in hepatocellular carcinoma cancer-associated fibroblasts affects cancer cell phenotypes and tumor growth
Source: Cell Oncol (Dordr). 2022 Nov 26;45(6):1435–49. doi: 10.1007/s13402-022-00730-4 (PMC9747837; doi:10.1007/s13402-022-00730-4)
Supplement: Supplementary file 4 — (DOCX 18 kb) [file 13402_2022_730_MOESM3_ESM.docx]

Table S1 Primers sequence

| Name | forward | reverse |
| --- | --- | --- |
| RT-PCR  LINC00152 | GCCCCCTCCAGCACCTCTACCT | GAAACCGACCAGACCAGCCCAT |
| RT-PCR  MIR100HG | CCACATTGGTCCACTTGACACT | CCCCTGAACTCATCTTTTGCTT |
| RT-PCR  Ki-67 | ACGCCTGGTTACTATCAAAAGG | CAGACCCATTTACTTGTGTTGGA |
| RT-PCR  PCNA | CCTGCTGGGATATTAGCTCCA | CAGCGGTAGGTGTCGAAGC |
| RT-PCR  CXCL11 | GACGCTGTCTTTGCATAGGC | GGATTTAGGCATCGTTGTCCTTT |
| RT-PCR  MiR-205-5p | RT:GTCGTATCCAGTGCGTGTCGTGGAGTCGGCAATTGCACTGGATACGACCAGACT  F: TCCUTCATTCCACCGG | CAGTGCGTGTCGTGGA |
| AGO2 RT-PCR  CXCL11 3’UTR | CAGTTATGTGAAGGATGAAAGGTGG | GCAACAAGTAAGAACGTGAAAGCA |
| AGO2 RT-PCR  LINC00152 | GTTCCAATGAGAATGAAGGCTGAGG | AAGCAGCGACCATCCAGTCATTTAT |
| RT-PCR  U6 | CTCGCTTCGGCAGCACA | AACGCTTCACGAATTTGCGT |
| RT-PCR  β-actin | TTCCAGCCTTCCTTCCTGGG | TTGCGCTCAGGAGGAGCAAT |
| sh-NC | GATCCGCCAAGCCCTTGTCTTCACAACTCGAGTTGTGAAGACAAGGGCTTGGCTTTTTG | AATTCAAAAAGCCAAGCCCTTGTCTTCACAACTCGAGTTGTGAAGACAAGGGCTTGGCG |
| Sh-LINC00152 1# | GATCCGACTCTGAGGCCTCTGCATTTCTCGAGAAATGCAGAGGCCTCAGAGTCTTTTTG | AATTCAAAAAGACTCTGAGGCCTCTGCATTTCTCGAGAAATGCAGAGGCCTCAGAGTCG |
| Sh-LINC00152 2# | GATCCGGTCTGGTCGGTTTCCCATTTCTCGAGAAATGGGAAACCGACCAGACC TTTTTG | AATTCAAAAAGGTCTGGTCGGTTTCCCATTTCTCGAGAAATGGGAAACCGACCAGACC G |
| LINC00152 overexpression | ctagcgtttaaacttaagcttTCGTTCCAATGAGAATGAAGGC | tgctggatatctgcagaattcTTTTTTTTTTTTTCTGTTTTCTTTAGTTT |
| Overexpression CXCL11 | ctagcgtttaaacttaagcttATGAGTGTGAAGGGCATGGC | tgctggatatctgcagaattcTTAAAAATTCTTTCTTTCAACTTTTTTGA |
| Sh-CXCL11 | GATCCGCCTCCATAATGTACCCAAGTCTCGAGACTTGGGTACATTATGGAGGCTTTTTG | AATTCAAAAAGCCTCCATAATGTACCCAAGTCTCGAGACTTGGGTACATTATGGAGGCG |
| Inhibitor NC | CAGUACUUUUGUGUAGUACAA |  |
| Mimics NC | UUCUCCGAACGUGUCACGUTT | ACGUGACACGUUCGGAGAATT |
| MiR-205-5p mimics | UCCUUCAUUCCACCGGAGUCUG | GACTCCGGTGGAATGAGGAUU |
| MiR-205-5p inhibitor | CAGACTCCGGTGGAATGAGGA |  |
| Wt-CXCL11 3’UTR | aattctaggcgatcgctcgagAAAAATATCAAAACATATGAAGTCCTGG | attttattgcggccagcggccgcAACTCCGATGGTAACCAGCCT |
| mut-CXCL11 3’UTR | GtagggaaTGAAAGGTGGGTGAAAGGACCAAA | CACCTTTCAttccctaCATAACTGTACAAAAGTTGAAAGTCACAA |
| Wt-linc00152 | TCGAGTCGTTCCAATGAGAATGAAGGCTGAGGTGTGCGCCTTTGGTACCGC | GGCCGCGGTACCAAAGGCGCACACCTCAGCCTTCATTCTCATTGGAACGAC |
| Mut-linc00152 | TCGAGTCGTTCCAATGAGGAATGAAGCTGAGGTGTGCGCCTTTGGTACCGC | GGCCGCGGTACCAAAGGCGCACACCTCAGCTTCATTCCTCATTGGAACGAC |
| LINC00152 probe  Biotin labeled | GGGCTCAGGCACCGCTTGTCTGGAATGTCA |  |
| MiR-205-5p probe  DIG labeled | CAGACTCCGGTGGAATGAAGGA |  |
